# Pseudomonas fragariae sp. nov., a novel bacterial species causing leaf spots on strawberry (Fragaria×ananassa)

**DOI:** 10.1099/ijsem.0.006476

**Published:** 2024-08-14

**Authors:** Marcus Vinicius Marin, Renato Carvalho, Mathews L. Paret, Jeffrey B. Jones, Natalia A. Peres

**Affiliations:** 1 Department of Plant Pathology, University of Florida, Gulf Coast Research and Education Center, Wimauma, FL 33598, USA; 2 Plant Pathology Department, University of Florida, Gainesville, FL 32611, USA

**Keywords:** bacteria taxonomy, novel bacteria, strawberry, whole genome sequencing

## Abstract

In Florida, angular leaf spot, caused by *Xanthomonas fragariae*, was the only known bacterial disease in strawberry, which is sporadic and affects the foliage and calyx. However, from the 2019–2020 to 2023–2024 Florida strawberry seasons, unusual bacterial-like symptoms were observed in commercial farms, with reports of up to 30 % disease incidence. Typical lesions were water-soaked and angular in early stages that later became necrotic with a circular–ellipsoidal purple halo, and consistently yielded colonies resembling *Pseudomonas* on culture media. Strains were pathogenic on strawberry, fluorescent, oxidase- and arginine-dihydrolase-negative, elicited a hypersensitive reaction on tobacco, and lacked pectolytic activity. Although phenotypic assays, such as fatty acid methyl profiles and Biolog protocols, placed the strains into the *Pseudomonas* group, there was a low similarity at the species level. Further analysis using 16S rRNA genes, housekeeping genes, and whole genome sequencing showed that the strains cluster into the *Pseudomonas* group but do not share more than 95 % average nucleotide identity compared to representative members. Therefore, the genomic and phenotypic analysis confirm that the strains causing bacterial spot in strawberry represent a new plant pathogenic bacterial species for which we propose the name *Pseudomonas fragariae* sp. nov. with 20-417^T^ (17^T^=LMG 32456^T^=DSM 113340 ^T^) as the type strain, in relation to *Fragaria*×*ananassa*, the plant species from which the pathogen was first isolated. Future work is needed to assess the epidemiology, cultivar susceptibility, chemical sensitivity, and disease management of this possible new emerging strawberry pathogen.

*Pseudomonas* is a highly diverse bacterial genus that includes members that cause disease in humans, animals, and plants [1,4]. To date, there are more than 330 validly published *Pseudomonas* species in the genus and the number is still increasing [5,7]. The development of novel approaches to distinguish prokaryotes has helped to increase and support the accuracy of traditional phenotypic assays used for many years for characterizing bacteria [8,12]. For example, advances in genomic sequencing technologies and the availability of databases useful for comparisons can be used to infer phylogenetic relationships in bacteria. Moreover, whole genome sequencing has enabled calculations of average nucleotide identity (ANI) or genome-to-genome distances, which can replace the laborious gold standard DNA–DNA hybridization (DDH) assay to elucidate bacterial relationships [13,19]. Methods such as DDH were used to assess the similarity among species, in which strains with over 70 % DDH were members of the same species [20]. Although DDH based on laboratory experiments became the benchmark for identifying bacterial species, its application was not widespread due to challenges such as the inability to create a database and the difficulty in obtaining reproducible experiments [14,16, 19]. In a previous study, an ANI of 95 % (± 0.5 %) was determined to approximate 70 % similarity based on DDH, and has served as a threshold for defining a new prokaryotic species [17]. Therefore, sophisticated molecular approaches combined with traditional phenotypic assays can result in an accurate and reliable pipeline to differentiate strains and new species. As plant pathogens, *Pseudomonas* species can cause disease in an array of hosts [21,23]. The species complex of *Pseudomonas syringae*, for example, has the largest group of pathovars causing disease in monocots, dicots, and woody plants [24,27].

The cultivated strawberry (*Fragaria* ×*ananassa*) is a high-value specialty crop popular for its colour, taste, and health benefits, such as vitamins and antioxidants [28]. In 2020, the United States strawberry production was about 1.1 million tonnes, with a total value of approximately US$2.6 billion [29]. California is the leading state responsible for about 90 % of the strawberry production; however, Florida is the largest winter producer with about 89 thousand tonnes over its 4000 harvested hectares, raising US$240 million in 2020 [30]. In Florida, the most common strawberry diseases are primarily caused by fungi and oomycetes, including anthracnose fruit rot (*Colletotrichum acutatum*), grey mould (*Botrytis cinerea*), powdery mildew (*Podosphaera aphanis*), crow rot (*Phytophthora cactorum*, *Colletotrichum gloeosporioides*, and *Macrophomina phaseolina*), and pestalotia leaf spot and fruit rot (*Neopestalotiopsis* sp.) [31,36]. The only known disease caused by a bacterium is angular leaf spot (ALS), caused by *Xanthomonas fragariae*, which affects the foliage and calyx, and occurs sporadically across different seasons, being particularly problematic during rainy and wet weather or when overhead irrigation is used for freeze protection [37,38]. Typical symptoms of ALS are semitranslucent angular lesions with water-soaked appearance that eventually becomes necrotic producing reddish-brown and irregular spots on the upper leaf surface and calyx [39]. However, beginning in the 2019–2020 Florida strawberry season and continuing through the 2023–2024 season, unusual bacterial-like lesions were observed in commercial farms, with reports of up to 30 % disease incidence on the newly released cultivar ‘Medusa’. On the upper and lower leaf surfaces, lesions had water-soaked and angular appearance in early stages that later became necrotic with a circular to ellipsoidal purple halo (Fig. 1). Isolations were carried out by surface sterilizing the entire leaflet for 1 min in a solution of 0.3 % NaOCl plus 0.05 % Tween 20. Afterward, symptomatic but not necrotic tissue pieces (~1 mm^2^) were collected, individually smashed in a droplet of deionized sterile water, and streaked on Wilbrink’s medium [40]. Isolation plates were incubated at 25 °C and 12 : 12 h of photoperiod. After 48 h, white, mucoid bacterial colonies resembling *Pseudomonas* were consistently observed. Single colonies from the isolation plates were streaked at least twice on nutrient agar (NA) medium for purification, then saved in the culture collection of the Strawberry pathology (Wimauma, FL) and Bacteriology laboratory (Gainesville, FL) of the University of Florida. A representative isolate was also deposited in DSMZ-Deutsche Sammlung von Mikroorganismen und Zellkulturen GmbH, and BCCM/LMG Bacteria collection.

**Fig. 1.**
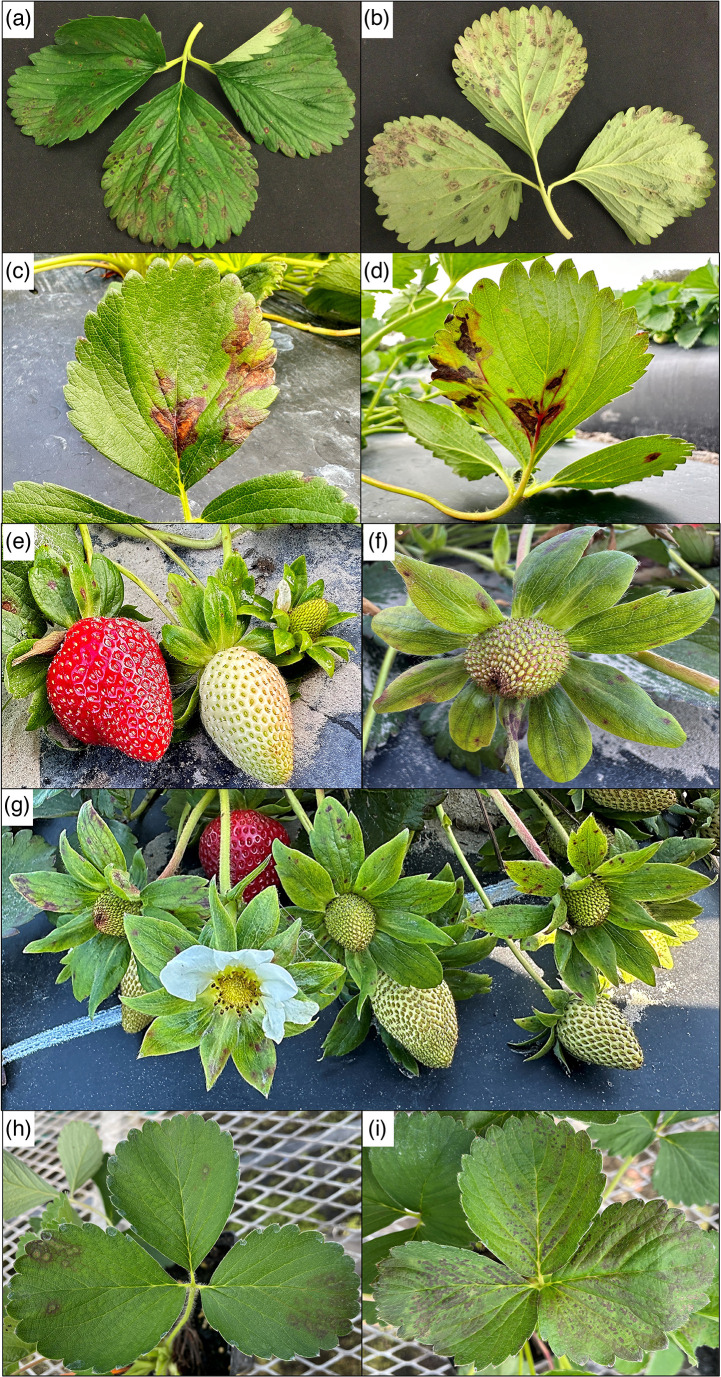
Original symptoms of small necrotic spots with purple halo (**a and b**), coalescing leaf spots (**c and d**), and spotting on calix (E to G) caused by *Pseudomonas fragariae* observed on ‘Medusa’. Symptoms developed on Sensation^®^ ‘Florida127’ during pathogenicity tests at 4 (**h**) and 10 (**i**) days after inoculation, confirming *P. fragariae* as the causal agent of the disease.

For the pathogenicity assay, three representative isolates were grown on NA medium and 24-hour-old plates used for inoculum preparation. A solution of sterile deionized water containing 0.1 % Tween 20 as a dispersing agent and 2.5 g l^−1^ MgSO_4_·7H_2_O was used for preparing bacterial suspensions. Inoculum concentration was determined using a Nanodrop and standardized to 800 nm, which was approximately 10^8^ c.f.u. ml^−1^. Four 1-month-old potted plants of each cultivar Sensation^®^ Florida127 and Florida Brillance were used per treatment, and the experiment was repeated three times. Treatments consisted of three isolates plus a non-inoculated control. Inoculations were performed by spraying the bacterial suspension using a spray bottle, until run-off. The non-inoculated controls were sprayed with the same solution containing Tween and MgSO_4_·7H_2_O that was used for inoculum preparation. After inoculation, plants were covered with clear plastic boxes for 48 h. Afterward, plants were kept in a greenhouse under mists that were set to be on for 6 s every 30 min during the day. On inoculated plants, symptoms similar to the original lesions were initially observed 4 days after inoculation; the disease continued to develop and became quite severe after 10 days, on both cultivars (Fig. 1). Throughout the experiments, the non-inoculated plants remained healthy. Re-isolations from symptomatic tissues were carried out and the same bacterium was observed. Analysis of the 16S rRNA genes of both original strains and strains recovered after inoculation had 100 % identity confirming the disease was caused by the inoculated strains.

In order to identify the bacteria isolated from strawberry, initial analysis such as a KOH test showed that the bacterium was Gram-negative. In addition, the strains produced a fluorescent pigment under ultraviolet light when grown on King’s medium B, indicating that the bacteria could belong to the genus *Pseudomonas*. Phylogenetic analysis such as 16S rRNA gene and multi-locus sequence analysis (MLSA) were performed. To sequence the genes, genomic DNA was extracted following the guidelines of the Wizard Genomic DNA Purification kit (Promega) and used as DNA template. First, 16S rRNA genes were amplified from DNA templates using the primers described by Pei *et al.* [41]. Moreover, the portions of genes *gyrB*, *rpoB*, and *rpoD* were amplified using the primers described by Agaras *et al*. [42], Ait Tayeb *et al*. [43], and Mulet *et al*. [44], respectively. Amplicons were purified following the guidelines of Qiagen PCR purification kit and sent to Eurofins Genomics (Louisville, KY) for sequencing. Sequences were used for comparisons in GenBank using a blastn query to distinguish genera and determine similarity to published type strains. Moreover, the sequences were used to reconstruct phylogenetic trees to identify similarities to bacterial type strains The 16S rRNA sequence analysis placed the strains into the genus *Pseudomonas* (Fig. S1, available in the online Supplementary Material). Further analysis of a phylogenetic tree with the concatenated *gyrB, rpoD*, and *rpoB* genes supported the results from the 16S rRNA gene analysis showing a close relation with *Pseudomonas* species. Although both 16S rRNA gene analysis and MLSA indicated a close relationship between the strains isolated from strawberries and members of *Pseudomonas*, such as *Pseudomonas syringae* pv. *aceris*, the results suggested that these strains might belong to a new species (Fig. 2). Following the above genomic analysis for species differentiation, the strains were subjected to whole genome sequencing and calculation of ANI. Bacteria were grown overnight in nutrient broth and the genomic DNA was extracted as mentioned above. The samples were sequenced by the Microbial Sequencing Center (Migs, Pittsburgh PA) using a single library preparation method based upon Illumina MiSeq 2×150 base pairs sequencing. Using Trim Galore (version 0.6.3) with default settings, adapters were trimmed and raw fastq reads were paired. The paired-end sequences were assembled into contigs using a pipeline based on the SPAdes assembler version 3.10.1 [45,46]. Contigs were generated using k-mers of 21, 33, 55, 77, 99, and 127, and any contigs smaller than 500 bp with a k-mer coverage less than 2.0 were discarded. The assembled genomes were uploaded to jSpecies (https://imedea.uib-csic.es/jspecies/about.html) to calculate the percentage of ANI based on the blast algorithm (ANIb). First, the contigs were used to calculate a pairwise ANIb) and then used to calculate comparisons within validly published *Pseudomonas* species that were downloaded from the National Center for Biotechnology Information (www.ncbi.nlm.nih.gov). A web-based tool, namely the Genome-to-Genome Distance Calculator version 3.0 (https://ggdc.dsmz.de/ggdc.php [47], was used to compute the *in silico* DDH between the novel species 20-417 (strain 17^T^) and the closest published reference strain based on ANI. Based on previous studies, species that share more than 95 % ANIb (±0.5 %) are related to 70 % of DDH and are considered the threshold to species differentiation in prokaryotic species [17]. The results of pairwise ANIb confirmed that the five isolates from strawberry are the same species (>99 % ANIb), and the closest type strain is *P. syringae* DSM 10604^T^ (<95 % ANIb). Moreover, the species does not share more than 95 % ANIb with validly published species in the *Pseudomonas* group [48] (Table 1). Along with ANIb, the *in silico* DDH estimated for 20-417^T^ (strain 17^T^) and the *P. syringae* DSM 10604^T^ was about 60 % (57.6–63.3 %), significantly below the 70 % threshold value recommended for species delineation.

**Fig. 2.**
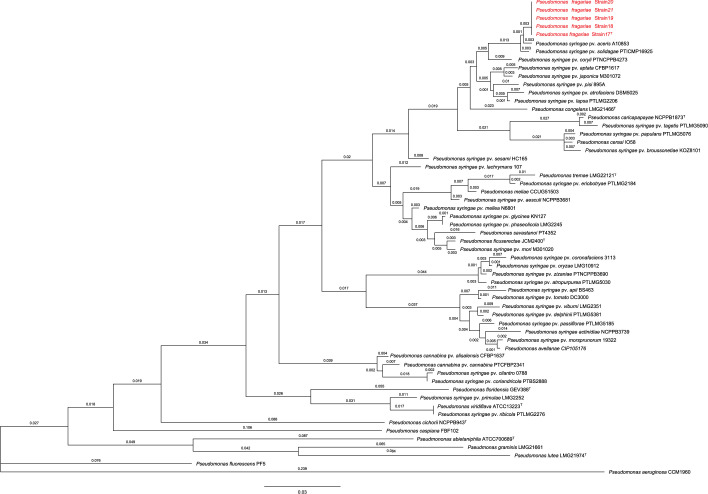
Maximum-likelihood analysis based on the concatenated alignments consisting of the housekeeping genes *rpoB*, *rpoD*, and *gyrB*. The strains isolated from strawberry, labelled as *Pseudomonas fragariae* strain 17^T^ to 21, were compared with members of the genus *Pseudomonas*. The type strain is *Pseudomonas fragariae* strain 17^T^. Bootstrap values are indicated in the clades.

**Table 1.** Average nucleotide identity–blast (ANIb) calculated with the whole genome sequences of the strains isolated from strawberry and compared with validly published members of the genus *Pseudomonas* Strains: 1, 17^T^; 2, 18; 3, 19; 4, 20; 5, 21; 6, *P. syringae* DSM 10604^T^; 7, *P. syringae* pv. *aceris* ICMP 9851; 8, *P. syringae* pv. *atrofaciens* LMG5092; 9, *P. syringae* pv. *aptata* G733; 10, *P. amygdali* CFBP 3205; 11, *P. syringae* pv. *actinidiae* CFBP1392; 12, *P. syringae* pv. *meliae* CFBP3225; 13, *P. syringae* pv. *primulae* ICMP3956; 14, *P. asturiensis* LMG 26898^T^; 15, *P. ficuserectae* ICMP 7848^T^; 16, *P. syringae* pv. *delphinii* ICMP529; 17, *P. savastanoi* ICMP 4352^T^; 18, *P. tremae* ICMP 9151^T^; 19, *P. viridiflava* CFBP 2107^T^; 20, *P. coronafaciens* CFBP 2216^T^; 21, *P. syringae* pv. *lachymans* ICMP448; 22, *P. beberidis* ICMP 4116^T^; 23, *P. floridensis* GEV358^T^; 24, *P. avellanae* BPIC 631^T^; 25, *P. syringae* pv. *tomato* DC3000. Pairwise analysis among the five strains exhibits more than 95 % ANIb, indicating that the five strains are the same species. Moreover, the strains isolated from strawberry does not share more than 95 % ANIb with other members in the *Pseudomonas* group, indicating that the strains belong to a novel species.

Strain	1	2	3	4	5	6	7	8	9	10	11	12	13	14	15	16	17	18	19	20	21	22	23	24	25
**1**	–	99.99	100.00	99.95	99.99	94.93	94.20	94.94	94.28	88.80	86.73	89.03	82.95	82.67	88.73	86.48	88.88	85.10	82.96	85.18	88.69	86.21	82.90	86.41	86.22
**2**	99.99	–	99.99	99.96	99.99	94.95	94.20	94.92	94.30	88.83	86.72	89.05	82.96	82.69	88.71	86.51	88.92	85.13	83.01	85.20	88.72	86.26	82.98	86.42	86.25
**3**	99.99	99.99	–	99.96	100.00	94.92	94.24	94.92	94.28	88.82	86.69	89.04	82.95	82.70	88.72	86.47	88.90	85.11	83.01	85.17	88.70	86.23	82.96	86.37	86.21
**4**	99.92	99.92	99.92	–	99.92	94.90	94.19	94.86	94.28	88.85	86.54	89.03	83.01	82.75	88.77	86.30	88.89	85.13	83.04	85.25	88.72	86.24	82.74	86.39	86.25
**5**	100.00	99.99	99.99	99.96	–	94.91	94.22	94.92	94.28	88.83	86.71	89.04	82.96	82.70	88.72	86.47	88.90	85.13	83.02	85.20	88.69	86.25	82.95	86.41	86.23
**6**	94.98	94.99	94.99	94.97	94.98	–	94.18	98.02	94.37	88.64	86.54	88.84	82.71	82.63	88.58	86.30	88.72	84.90	82.76	85.01	88.67	86.13	82.79	86.25	86.08
**7**	94.21	94.21	94.21	94.20	94.21	94.19	–	94.41	98.90	89.09	86.33	89.13	82.89	82.82	89.12	86.17	89.14	85.00	82.79	85.21	89.01	86.11	82.67	86.22	86.11
**8**	94.78	94.78	94.78	94.75	94.78	97.75	94.15	–	94.27	88.64	86.56	88.92	82.71	82.63	88.65	86.12	88.78	84.88	82.69	85.07	88.47	86.01	82.88	86.07	85.92
**9**	94.37	94.37	94.37	94.37	94.38	94.39	98.90	94.50	–	89.19	86.33	89.30	82.87	82.79	89.12	86.15	89.29	85.03	82.86	85.11	89.08	86.15	82.68	86.22	86.11
**10**	88.97	88.97	88.97	88.99	88.97	88.75	89.19	88.79	89.31	–	87.32	98.35	82.88	82.80	97.46	87.20	98.81	85.49	82.80	85.38	97.58	87.22	82.55	87.46	87.21
**11**	86.84	86.84	86.84	86.72	86.83	86.57	86.52	86.58	86.44	87.27	–	87.48	83.38	83.20	87.26	96.95	87.38	86.12	83.28	85.99	87.40	95.23	83.34	97.49	95.33
**12**	89.10	89.10	89.10	89.12	89.10	88.89	89.31	88.95	89.42	98.40	87.44	–	83.13	83.03	97.55	87.44	98.51	85.72	83.09	85.54	97.74	87.39	82.88	87.58	87.38
**13**	83.21	83.21	83.21	83.21	83.21	82.88	83.00	83.00	83.01	82.90	83.31	83.12	–	86.94	82.80	83.31	82.88	82.41	96.34	82.41	82.90	83.06	86.29	83.39	83.21
**14**	82.95	82.95	82.95	83.00	82.96	82.79	82.94	82.86	82.94	82.81	83.20	82.99	86.93	–	82.76	83.16	82.84	82.29	86.91	82.32	82.79	82.97	85.69	83.21	83.15
**15**	88.85	88.86	88.85	88.93	88.85	88.62	89.21	88.68	89.22	97.39	87.29	97.40	82.97	82.83	–	87.20	97.44	85.45	82.93	85.35	97.91	87.13	82.64	87.41	87.16
**16**	86.73	86.73	86.74	86.60	86.74	86.48	86.37	86.45	86.32	87.15	96.90	87.45	83.30	83.11	87.09	–	87.24	86.10	83.21	86.04	87.22	95.79	83.21	97.00	95.96
**17**	88.93	88.93	88.93	88.92	88.93	88.62	89.14	88.71	89.27	98.69	87.35	98.38	82.89	82.85	97.43	87.22	–	85.52	82.86	85.46	97.64	87.27	82.58	87.46	87.27
**18**	85.41	85.41	85.41	85.42	85.40	85.14	85.23	85.23	85.29	86.21	86.01	86.32	82.35	82.19	86.04	85.98	86.31	–	82.34	98.16	86.15	86.40	82.08	86.21	86.31
**19**	83.19	83.19	83.19	83.20	83.19	82.89	82.91	82.92	82.95	82.86	83.23	83.11	96.37	86.91	82.82	83.22	82.87	82.43	–	82.44	82.88	83.04	86.10	83.34	83.20
**20**	85.37	85.37	85.36	85.43	85.36	85.07	85.26	85.15	85.24	85.46	86.18	85.55	82.52	82.40	85.34	86.10	85.52	98.85	82.52	–	85.48	86.39	82.24	86.35	86.23
**21**	88.92	88.92	88.93	88.94	88.92	88.81	89.09	88.69	89.19	97.53	87.41	97.54	82.95	82.84	97.85	87.30	97.65	85.46	82.89	85.41	–	87.42	82.67	87.53	87.44
**22**	86.47	86.46	86.46	86.48	86.46	86.27	86.26	86.25	86.30	87.23	95.24	87.43	83.00	82.92	87.06	95.78	87.25	86.40	82.93	86.29	87.38	–	82.76	95.57	98.72
**23**	83.17	83.17	83.17	82.96	83.17	82.93	82.77	83.08	82.83	82.58	83.26	82.86	86.27	85.65	82.51	83.19	82.55	82.14	86.14	82.17	82.61	82.78	–	83.05	82.90
**24**	86.73	86.73	86.73	86.73	86.73	86.49	86.42	86.45	86.54	87.49	97.40	87.61	83.45	83.36	87.45	96.95	87.58	86.41	83.43	86.38	87.62	95.59	83.19	–	95.50
**25**	86.48	86.48	86.48	86.49	86.48	86.28	86.28	86.23	86.30	87.11	95.12	87.37	83.14	83.06	87.13	95.71	87.33	86.34	83.08	86.15	87.60	98.73	82.95	95.47	–

To confirm the novel species, several phenotypic assays were performed with the strains. Among the assays, LOPAT tests, which stands for levan production, oxidase reaction, pectolytic activity, arginine di-hydrolase activity and tobacco hypersensitivity, are traditionally used for pseudomonads differentiation [49]. Levan production was tested by growing bacteria in NA amended with 5 % sucrose. All the strains showed positive production of levan (a mucoid white material) indicating that the bacteria contain levansucrase enzyme able to break down the sucrose present in the media. The oxidase test was performed by smearing bacterial growth on slides containing tetramethyl-*p*-phenylenediamine di-hydrochloride. All the colonies showed a late development of colour (more than 15 s) indicating a negative result of oxidase activity. The pectolytic activity assay was determined by streaking bacterial cells on crystal violate pectate medium. None of the samples showed the development of pitting on the bacteria indicating a negative result for pectolytic activity. Arginine dihydrolase activity was tested by inoculating bacterial suspensions in tubes containing Thornley’s medium adjusted to pH 7.2. After incubating for 4 days at 28 °C, none of the strains showed colour change indicating a negative result for arginine dihydrolase activity. Finally, a hypersensitive response (HR) in tobacco was tested by inoculating a fresh bacterial suspension (10^8^ c.f.u. ml^−1^) into the mesophyll of tobacco leaves. The inoculated plants were incubated at 28 °C and the formation of confluent necrosis at the infiltration site after 24 h indicated positive results for HR in tobacco. The LOPAT results of the strains isolated from strawberry are similar to results of *P. syringae* pv. *tomato* (DC3000). Moreover, the results differ from other members of *Pseudomonas* such as *P. viridiflava* ICMP 2848^T^ which shows a positive reaction in the pectolytic activity. Moreover, *P. cichorii* ATCC 10857^T^ produced a positive oxidase reaction differing from the strains isolated from strawberry. Finally, *P. quasicaspiana* CDFA553^T^ produced a negative HR in tobacco unlike the strawberry strains (Table 2).

**Table 2.** LOPAT results for the strain isolated from strawberry and comparisons with validly published species in the *Pseudomonas* group Strains: 1, 17^T^ (this study); 2, *P. syringae* pv. *tomato* DC3000; 3, *P. viridiflava* ICMP 2848^T^ [53]; 4, *P. cichorii* ATCC 10857^T^ [54]; 5, *P. quasicaspiana* CDFA553 [5]. All five strains of *Pseudomonas fragariae* in this study showed the same reactions.

Test	1	2	3	4	5
Levan production	+	+	−	−	−
Oxidase activity	−	−	−	+	+
Pectolytic activity	−	−	+	−	+
Arginine dihydrolase activity	−		−	−	−
HR in tobacco	+	+	+	+	−

The next assay aiming to distinguish the bacteria was the Biolog system, which identifies carbon-source fingerprints, reaction with different salts, and pH preferences/tolerance. For this assay, we used the GEN III MicroPlate according to the manufacturer’s instructions. The Biolog system is useful for identifying bacterial reactions and also compares the results with a large databank to distinguish bacterial species. For the Biolog tests, the strains were grown overnight in bacterial universal growth media and resuspended in Biolog inoculation fluid at a final turbidity of 95 %. One hundred microlitres of the inoculated fluid were added to each well in a Biolog GENIII MicroPlate, then the plates were incubated at 28 °C for 48 h, and read using a Biolog reader. Although the fingerprint results placed the bacterial samples into the genus *Pseudomonas*, the results showed low similarity at species level. Interestingly, the results for the novel strains differed from those of their closely related species; for example, the pectin result was positive for the strawberry strains and negative for *P. syringae* pv. *tomato*. A similar trend was observed in terms of the l-lactic acid reaction results, which were positive for the strawberry strains and negative for *P. syringae* pv. *tomato* (DC3000). Moreover, the acetoacetic acid and sodium butyrate reactions were positive for the strawberry strains, but negative for *P. quasicaspiana* (CDFA553) and *P. syringae* pv. *tomato* (DC3000).

Following the phenotypic assays, the strains were subjected to fatty acid methyl profile (FAME) analysis. Each bacterial strain was grown to exponential phase (24 h) on trypticase soy agar. Bacterial cells were harvested and fatty acids were extracted according to the standard midi protocol [50]. FAME analysis was done using the Sherlock Microbial Identification System 4.5 [51]. FAME analysis has been useful for distinguishing bacteria by quantitatively and qualitatively analysing key components of bacterial membranes, such as phospholipids [52]. To profile the FAMEs of the novel bacteria, cells were sent for identification using the Microbial Identification System (midi, Newark, DE). Significant peaks in the profiles of the strawberry strains included summed feature 3 (C_16 : 1_ ω7*c*/C_16 : 1_ ω6*c*), saturated C_16 : 0_, and summed feature 8 (C_18 : 1_ ω7*c*). The similarity between the sample isolated from strawberries and the FAME databank indicated an index of 0.797 with *Pseudomonas syringae* pv. *lachrymans*.

To determine the morphological characteristics of the strawberry strains, bacterial cells were subjected to transmission electron microscopy (TEM). The bacterial suspension was prepared using 24 h fresh cells grown in nutrient broth and the samples were submitted to negative stain imaging at the Interdisciplinary Center for Biotechnology Research (ICBR – University of Florida, Gainesville). A 400-mesh carbon-coated Formvar nickel grid was glow discharged using pelco easiGlow (Ted Pella) followed by floating onto a 10 ul droplet of the sample for 5 min; excess solution was drawn off with filter paper. The sample grid was then floated on a 10 ul droplet of 2 % aqueous uranyl acetate for 30 s, stains removed with filter paper, air dried, and examined with an FEI Tecnai G2 Spirit Twin TEM. Digital images were acquired with a Gatan UltraScan 2k x 2k camera and Digital Micrograph software (Gatan). TEM images showed a rod-shaped cell measuring about 2–2.5 µm long and 1 µm wide with a lophoritrichous flagellar arrangement (Fig. 3).

**Fig. 3.**
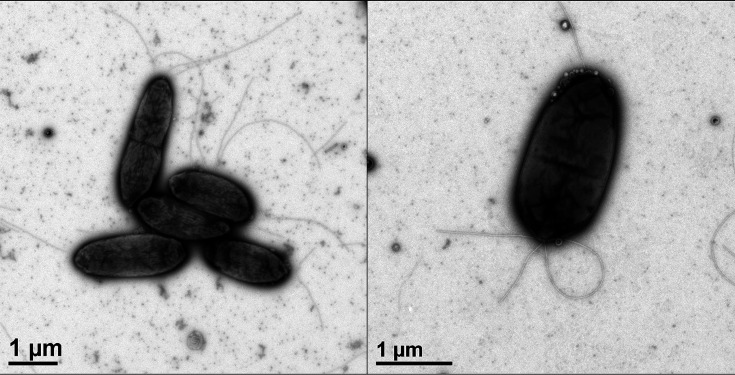
Transmission electron microscopy of the bacterial cells of the strains isolated from strawberry. Cells are rod-shaped cell measuring about 2–2.5 µm long and 1 µm wide showing a lophotrichous flagellar arrangement.

In summary, the pathogenicity tests showed that the strains isolated from strawberry are plant-pathogenic bacterial strains. The genomic analyses, including sequence analysis of 16S rRNA genes, MLSA using housekeeping genes, and ANI calculated with the whole genome, confirmed that the strains belong to a new species of plant-pathogenic bacteria belonging to the genus *Pseudomonas*. Moreover, the results were supported by a series of phenotypic and morphological assays. Therefore, a novel species name *Pseudomonas fragariae* sp. nov. is proposed.

## Description of *Pseudomonas fragariae* sp. nov.

*Pseudomonas fragariae* (*fra.ga’ri.ae*. L. gen. n. *fragariae*, pertaining to *Fragaria* the host genus where the strains were first isolated).

The cells are Gram-negative, rod-shaped (2–2.5 µm long and 1 µm wide), and exhibit a lophotrichous flagellar arrangement. They are fluorescent on King’s medium B agar and are oxidase-negative. The colonies are smooth and circular in shape, 0.5–1.0 mm diameter after incubation in 28 °C for 24 h on NA medium. Strains are levan-positive, oxidase-negative, and arginine-dihydrolase-negative. They are negative for pectinolytic activity and elicit an HR in tobacco. Cell growth occurs between 4 and 37 °C, with optimum growth observed between 26 and 29 °C. The bacterium grows at pH 5 and 8 and in broth containing up to 4 % NaCl (w/v). Cells are positive for the utilization of α-d-glucose, d-frutose, d-galactose, inosine, 1 % sodium lactate, fusidic acid, d-serine, d-arabitol, inositol, glycerol, d-serine, troleandomycin, minocycline, glycyl-l-proline, l-alamine, l-arginine, l-aspartic acid, l-glutmic acid, l-histidine, l-serine, lincomycin, guanidine HCl, Niaproof 4, pectin, d-galacturonic acid, l-galacturonic acid lactine, d-gluconic acid, d-glucuronic acid, mucic acid, quinic acid, d-saccharic acid, vancomycin, tetrazolium violet, tetrazolium blue, l-latic acid, citric acid, α-keto-glutaric acid, d-malic acid, l-malic acid, nalidixic acid, lithium chloride, potassium tellurite, Tween 40, γ-amino-brutryric acid, β-hydroxy-d,l-butyric acid, α-keto-butyric acid, acetoacetic acid, propionic acid, acetic acid, formic acid, aztreonam, and sodm butyrate. Cells are negative for the utilization of dextrin, maltose, trehalose, cellobiose, cellobiose, gentiobiose, turanose, stachyose, raffinose, lactose, melibiose, methyl β-d-glucoside, d-salicin, *N*-acetyl-d-glucosamine, *N*-acetyl-β-d-mannosamine, *N*-acetyl-d-galatosamine, *N*-acetyl neuraminic acid, 3-methyl glucose, l-fucose, l-rhamnose, d-glucose-6-po4, d-fructose-6-PO_4_, d-aspartic acid, gelatin, l-pyroglutamic acid, *p*-hydroxy-phenylacetic acid, d-lactic acid methyl ester, and α-hydroxy-butyric acid. Major fatty acids are C_16 : 1_ ω7*c*/C_16 : 1_ ω6*c* (summed feature 3), saturated C_16 : 0_, and C_18 : 1_ ω7*c* (summed feature 8).

The type strain, 20-417^T^ (strain 17^T^=LMG 32456^T^=DSM 113340^T^), was isolated from symptomatic strawberry (*Fragaria*×*ananassa*) in Florida. The DNA G+C content of the type strain is 58 mol%, based on a genome size of ~6.1 Mb. Whole genome GenBank accessions: JAWHKR000000000 (strain 17^T^), JAXHPV000000000 (20-418, strain 18), JAXCEY000000000 (20-419, strain 19), JAXCFZ000000000 (20-420, strain 20), JAXCGA000000000 (20-421, strain 21). GenBank accession numbers of 16S rRNA, *gyrB*, *rpoB*, and *rpoD* genes: OQ380626, PP589944, PP589949, and PP589954 (strain 17^T^), PP496055, PP589945, PP589950, and PP589955 (20-418, strain 18), PP496056, PP589946, PP589951, and PP589956 (20-419, strain 19), PP496057, PP589947, PP589952, and PP589957 (20-420, strain 20), and PP496058, PP589948, PP589953, and PP589958 (20-421, strain 21).

## supplementary material

10.1099/ijsem.0.006476 Uncited Fig. S1.

## Abbreviations

ALS: angular leaf spot
ANI: Average Nucleotide Identity
ANIb: ANI based on BLAST+
DDH: DNA–DNA hybridization
FAME: fatty acid methyl profile
HR: hypersensitive response
LOPAT: L, levan production; O, oxidase production; P, pectinolitic activity; A, arginine dihydrolase production; and T, tobacco hypersensibility
NA: Nutrient agar
TEM: transmission electronmicroscopy
