# Supplementary material for: Pseudomonas fragariae sp. nov., a novel bacterial species causing leaf spots on strawberry (Fragaria×ananassa)
Source: Int J Syst Evol Microbiol. 2024 Aug 14;74(8):006476. doi: 10.1099/ijsem.0.006476 (PMC11324255; doi:10.1099/ijsem.0.006476)
Supplement: Uncited Fig. S1. [file ijsem-74-06476-s001.pdf]

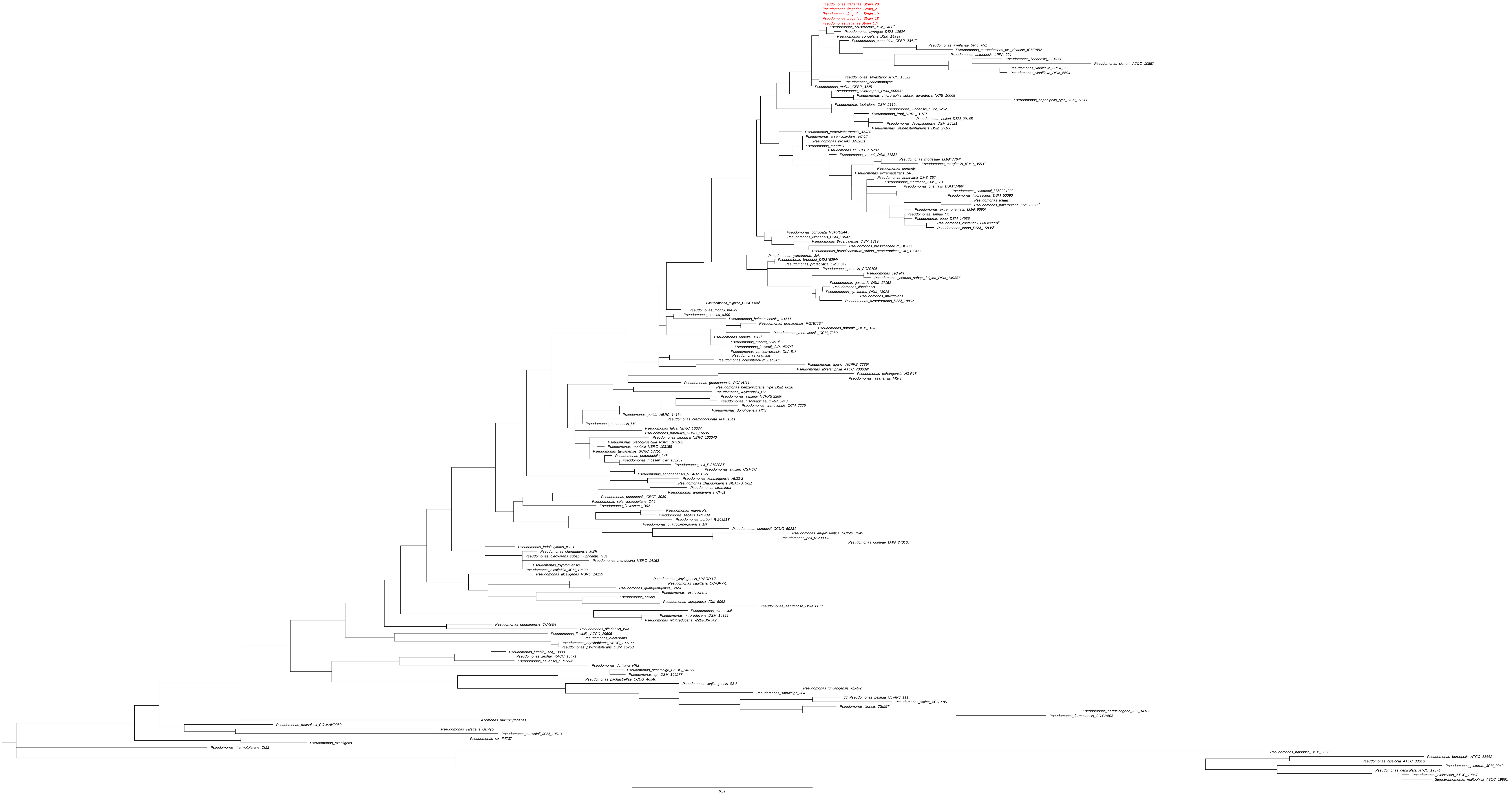

Supplemenary figure 1: Phylogenetic tree of 16 sRNA genes. The strains isolated from strawberry labeled a *Pseudomonas fragariae* strain 17^{T} to 21, were compared with members of the genus *Pseudomonas*.
